# Correlation of IOP with Corneal Acoustic Impedance in Porcine Eye Model

**DOI:** 10.1155/2017/2959717

**Published:** 2017-05-31

**Authors:** Jun Zhang, Yi Zhang, Yang Li, Ruimin Chen, K. Kirk Shung, Grace Richter, Qifa Zhou

**Affiliations:** ^1^College of Power and Mechanical Engineering, Wuhan University, Hubei, Wuhan 430072, China; ^2^Department of Biomedical Engineering, University of Southern California, Los Angeles, CA 90089, USA; ^3^USC Roski Eye Institute, Keck School of Medicine, University of Southern California, Los Angeles, CA 90033, USA

## Abstract

**Purpose:**

The aim of this study is to correlate the intraocular pressure (IOP) change with the acoustic impedance of the cornea, in order to propose a noncontact and noninvasive method for IOP monitoring.

**Methods and Materials:**

A highly focused transducer (frequency 47-MHz; bandwidth 62%) was made to measure the echo from the anterior and posterior surfaces of intact porcine eyes, respectively. A multilayered transmission and reflection model was used to calculate the acoustic impedance. The linear relationship between acoustic impedance and intraocular pressure was analyzed by statistical method.

**Result:**

During pressure elevation from 10 mm Hg to 50 mm Hg, the mean acoustic impedance of the posterior cornea increased from 1.5393 to 1.5698 MRayl, which showed a strong linear correlation (*R* = 0.9849; *P* = 0.0022). Meanwhile, the mean value of the anterior cornea increased from 1.5399 to 1.5519 MRayl, and a less significant correlation was observed (*R* = 0.7378; *P* = 0.0025).

**Conclusion:**

This study revealed a linear correlation between intraocular pressure and acoustic impedance of the cornea, thus demonstrating a potentially important method to noninvasively measure the intraocular pressure in vivo.

## 1. Introduction

Elevated intraocular pressure (IOP) is the main risk factor for glaucoma, which is a leading cause of irreversible optic nerve damage for blindness [1, 2]. Nowadays, several methods of IOP measurement have been developed for clinical use, including the gold-standard Goldmann applanation tonometer (GAT), the dynamic contour tonometer (DCT), Tono-Pen, and Icare rebound tonometer. A contact lens sensor (CLS) has also been developed for IOP monitoring [3–6], capable of providing data needed for the clinician to characterize certain types of glaucoma based on fluctuations in IOP for one 24-hour period. However, IOP measurements by some of the currently developed devices may be affected by ocular properties such as corneal curvature, hysteresis, and thickness [6, 7]. There is an unmet need for a noninvasive and accurate method for IOP measurement.

Previous works in ocular biomechanics have focused on studying the influence of intraocular pressure on the biomechanical properties of tissue such as cornea, sclera, and optic nerve. For example, strip extensiometry and inflation tests were always used for measurement of the cornea elastic modulus according to the nonlinear strain-stress curve [8]. However, due to different ocular conditions, there is inconsistency of the measured results in the prior studies [9–11]. Mathematical models were also established to improve the understanding of the complex biomechanical behavior of the eyeball. Anderson et al. used nonlinear finite-element modelling to study the behavior of the cornea under different loading states [12]. Elsheikh et al. used shell theory for pressure-deformation results analysis and found that the correlation between the modulus of elasticity in the cornea and intraocular pressure had a positive linear correlation, although the geometry deformation and stress change in the cornea were complex [13, 14]. This gives us a clue that we could use elasticity of the cornea or related parameters to characterize the IOP level.

Ultrasonic waves have been used to measure elastic properties of the cornea and sclera. Tanter et al. used supersonic shear waves to get three-dimensional quantitative maps of the corneal elasticity at two IOP levels (10 mmHg and 20 mmHg) [15–17]. Liu et al. used an ultrasound speckle tracking method to map 3D strains of the porcine sclera inflated from 15 mmHg to 19 mmHg [18–20]. However, these methods did not provide a direct relationship between the elastic modulus and intraocular pressure. Ultrasonic waves have also been used to measure the pressure in tissue directly. Eisenbrey et al. used a subharmonic aided pressure estimation (SHAPE) method to monitor portal hypertension in patients [21, 22]. Although this is a relatively noninvasive technique, a microbubble is still needed to generate a harmonic wave, and for the eye, this would require a surgical procedure to inject a bubble and thus would be rather invasive. Zhang et al. used a magnetic shaker to generate low frequency shear waves to estimate carpal tunnel pressure by measuring the shear wave speed in the tendon [23, 24]. The obstacle to apply this technique in the eye is that the shaker would vibrate the eye and potentially damage the delicate ocular structures. Recently, Liu et al. used a quantitative ultrasound spectroscopy method and the reflection amplitude method to measure the acoustic impedance of the canine cornea at a fixed intraocular pressure and found that the acoustic impedance has a linear relationship with the elastic modulus of the cornea, which increased nonlinearly with stress [25–27]. As mentioned above, the relationship of IOP and elasticity was also found to be linearly correlated. Theoretically, the correlation of IOP and corneal acoustic impedance could be achieved, and this relationship is expected to be linear. It is simple to characterize the IOP level directly based on the change of acoustic impedance, which is easily obtained by measuring the pulse echo of ultrasonic waves. So far, to the best of our knowledge, no study has reported on the relationship of IOP and corneal acoustic impedance.

In this study, we used a custom-made highly focused transducer (47 MHz, F# 0.9) to acquire the anteriorly and posteriorly reflected signal of cadaver porcine eyes at intraocular pressure ranging from 10 mmHg to 50 mmHg with an increasing interval of 10 mmHg. The reflected signals were used to calculate the acoustic impedance of the anterior and posterior segment of the cornea, which were based on reflection and transmission coefficient models established by continuous monitoring of stress and strain. Statistical analysis was used to correlate the acoustic impedance and the intraocular pressure.

## 2. Material and Method

### 2.1. Sample Preparation

Six fresh porcine eyes were collected from a local slaughterhouse and stored in a box at 0°C. The eyeball was place on a rubber pad with a hole in the center to maintain its position during inflation due to pressure elevation. Four pins were inserted into the extraocular muscles and the rubber pad to fix the eyeball. A 30-gauge needle connected with an infusion line was inserted though the limbus into the anterior chamber for IOP elevation. Balanced salt solution was used for both insertion into the chamber and immersion of the eyeball during ultrasound reflection testing. The IOP was decided by the height of saline bottle. The experimental temperature is 20°C. The same eye was measured three times with the same condition, and the mean values were used for analysis.

### 2.2. Transducers and System

**Figure 1:**
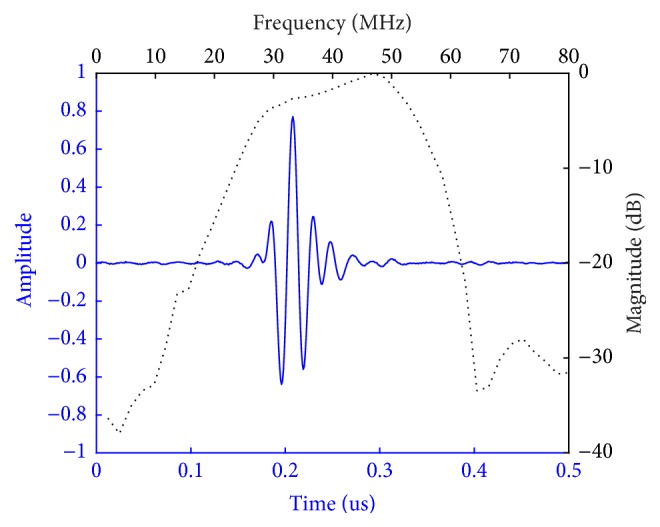
The pulse echo and frequency spectrum of the custom-made transducer.

A highly focused transducer was made to measure the echo from the anterior and posterior surfaces of the cornea, respectively. The frequency of the transducer is 47-MHz with a bandwidth of 62%. The element is 4 mm diameter and made of LiNbO_3_ (Boston Piezo-Optics, Bellingham, MA). Matching layers were used to acquire the large bandwidth. A steel ball with diameter of 7.2 mm was used to make foci of 3.6 mm. The pulse echo and frequency spectrum of the transducer are shown in Figure 1.

**Figure 2:**
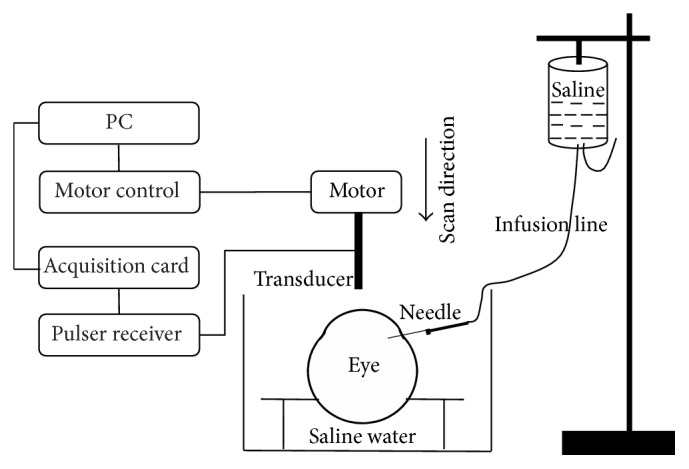
IOP measurement setup by ultrasound transducer.

The ultrasound system and pressure elevation setup are shown in Figure 2. The eyeball was fixed on a rubber holder. The transducer was aligned to the center of the eyeball by motor controller (ESP301, Newport) and scanned vertically with a step of 1 nm. The pulser/receiver (Panametrics 5900PR, Olympus, Waltham, MA) was set with a bandwidth from 1 MHz to 100 MHz to transmit and receive ultrasound signals. A high-speed acquisition card (GAGE) was used to acquire the signal with a sampling frequency of 1 GHz and 10 A-line scans and transmit it to personal computer via custom software. An infusion bottle with a needle was used to inject the balanced salt solution into the anterior chamber by the height difference between the bottleneck and eyeball. The height of the bottle could be adjusted to change the level of IOP.

### 2.3. Data Processing

To accurately adjust the transducer position to get the maximum pulse echo, one-dimensional scanning data was used to get the maximum amplitude and related time of flight by fitting of the acquired signals to a parabolic envelope curve.

Reflection coefficient models were used to calculate the acoustic impedance of the anterior and posterior cornea, respectively, and the highly focused transducer could completely separate the two reflected signals. For the anterior reflection, the reflection coefficient was only dependent on the acoustic impedance ratio of the immersion saline and anterior cornea. So the cornea impedance *Z*_1_ could be calculated by the following formula [27] when the impedance of saline *Z*_0_ is known:

(1) $$\begin{array}{c} \frac{A_{1}}{A_{0}}=\frac{Z_{1}-Z_{0}}{Z_{1}+Z_{0}}. \end{array}$$

*A*_1_ is the max amplitude of the reflected signal, while *A*_0_ is the amplitude of the incident signal. The echo of a quartz with known density and longitudinal wave velocity was used as the reference signal to calculate *A*_0_ by a similar formula as (1). As the reflection interface was not planar, the curvature coefficient was used for modification as mentioned in [28].

For posterior reflection, as Young's modulus of the anterior and posterior cornea proved to be different [29], the acoustic impedance was also considered separately. The saline, cornea, and anterior chamber could be considered as a three-layered medium. So the incident signal *A*_0_ and the reflected signal *A*_*r*_ has the following relationship:

(2) ArA0=T01R23T01,T01=2Z1Z1+Z0,  R23=Z3−Z2Z3+Z2,  T10=2Z0Z1+Z0.

*Z*_0_, *Z*_1_, *Z*_2_, and *Z*_3_ are the acoustic impedance of saline, anterior cornea, posterior cornea, and aqueous humor, respectively.

The Pearson correlation analysis [27] was used to correlate the corneal acoustic impedance and intraocular pressure. The correlation coefficients were calculated by functions provided by MATLAB Software.

## 3. Results

**Figure 3:**
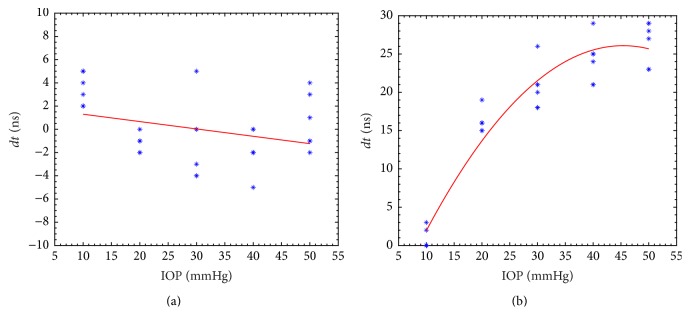
Difference of time of flight during pressure elevation for anterior cornea (a) and posterior cornea (b).

Figure 3 shows the time of flight difference at different intraocular pressures. For the anterior reflected signals, the difference of time that received the focused echo was limited by ±5 ns, which indicated that the experiment and data processing method exhibited good repeatability. For the posterior reflected signals, the difference of time of flight increased with IOP elevation, according to the fitted curve. There is an inconsistence that the thickness of the cornea would change or not with the IOP elevation [30, 31]. If the thickness of the cornea was not affected by changing IOP from 0 to 50 mmHg [30], the time of flight change is dominated by the velocity change of the cornea, which was induced by the elasticity change during pressure elevation. However, if the cornea thickness changed [31], the time of flight will affected both by velocity and thickness changing, which may lead to a nonlinear relationship as described in Figure 3(b). We cannot make a direct relationship between time of flight and IOP change due to complex deformation of eyeball. So we propose an acoustic impedance method which is not affected by the thickness change of cornea.

**Figure 4:**
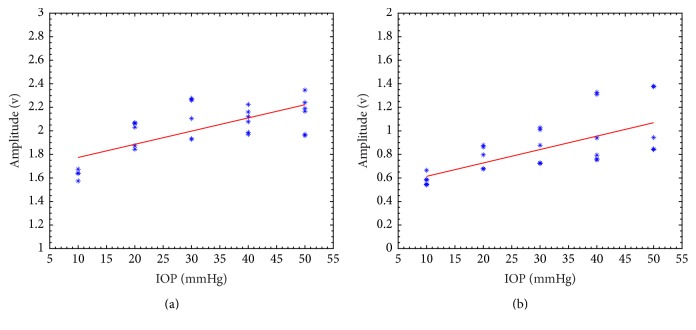
Amplitude change during pressure elevation for anterior cornea (a) and posterior cornea (b).

Figure 4 is the amplitude of the echo from the anterior and posterior interface. The dots represent the experimental amplitude of the echo as a function of the IOP level, while the solid line represents a linear fitting line. During the pressure elevation from 10 mm Hg to 50 mm Hg, the anterior amplitude increased from 1.6347 ± 0.0011 to 2.1454 ± 0.0234 and the posterior amplitude increased from 0.5778 ± 0.0022 to 1.0382 ± 0.0706. Strong correlation was observed (*R* = 0.9879; *P* = 0.0016) for the posterior amplitude with IOP, while less significant correlation was observed (*R* = 0.8651; *P* = 0.0581) for anterior amplitude.

**Figure 5:**
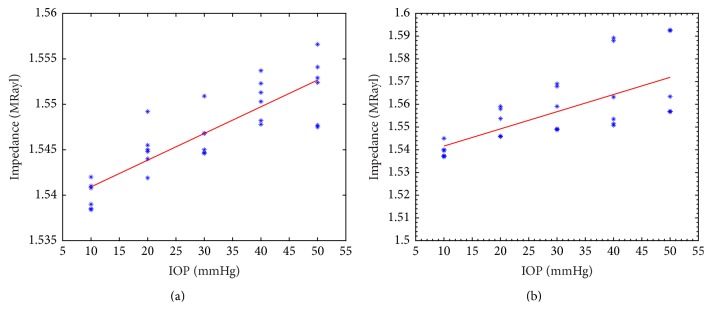
Cornea acoustic impedance during pressure elevation for anterior cornea (a) and posterior cornea (b).

Figure 5 is the acoustic impedance of the anterior and posterior cornea calculated by reflection model using the amplitude data in Figure 4. The dots represent the experimental acoustic impedance as a function of the IOP level, while the solid line represents a linearly fitted line. During the pressure elevation from 10 mm Hg to 50 mm Hg, the mean acoustic impedance of the anterior cornea increased from 1.5399 to 1.5519 MRayl. The mean value of posterior acoustic impedance increased from 1.5393 to 1.5698 MRayl. Strong correlation was observed (*R* = 0.9849; *P* = 0.0022) for the posterior acoustic impedance with IOP, while less significant correlation was observed (*R* = 0.7378; *P* = 0.0025) for anterior acoustic impedance.

## 4. Discussion

The measured corneal acoustic impedance showed a significant correlation with the intraocular pressure. However, data dispersion occurred due to the complex deformation behavior and elastic property change during pressure elevation.

For biomechanics analysis, the globe will get inflated as the intraocular pressure increases. Due to the irregular structure of the segments and their different properties, the deformation of cornea geometry, compression, and elasticity changes with IOP changes. According to Laplace law, the stress *σ* applied to the cornea is calculated using intraocular pressure *P*, cornea radius *R*, and cornea thickness *t*:

(3) $$\begin{array}{c} \sigma =\frac{PR}{2t}. \end{array}$$

The corneal thickness *t* would vary little or become thinner if the IOP changes in a reasonable range, as mentioned before [30, 31]. The corneal radius should increase during the inflation process, so according to (3), stress should increase during IOP elevation. The relationship between stress *σ* and strain *ε* for the cornea is nonlinear, which means that the elastic modulus *E* (secant or tangent) should increase as stress increases. The linear relationship of stress *σ* and elastic modulus *E* has been demonstrated by shell theory and prior experiments [14]. The matrix of the cornea is a viscoelastic and nearly incompressible material; this means that the density of the cornea should not vary during inflation [32]. According to the above discussion, IOP should have a direct effect on the acoustic impedance, which is the product of density and acoustic velocity. However, due to the complexity of the deformation process, an explicit expression of the relationship could not be found. Fortunately, the linear relationship between the acoustic impedance and elastic modulus of the cornea has been demonstrated experimentally in [27]. Thus, we can safely conclude that the IOP and cornea impedance has a linear relationship, which is also demonstrated in our experiments. However, data dispersion was observed. This may due to the assumptions and approximations we made, in spite of experimental error. For example, the change in cornea curvature, which will affect the reflection coefficient of incident ultrasound wave, was ignored.

It is important to accurately measure the acoustic impedance because a large difference in intraocular pressure could only induce a small change in impedance, as shown in Figure 5. Several technical methods should be taken to improve accuracy. Firstly, a precision device should be used to align the center of the ultrasonic transducer and the cornea for vertical scanning in an in vitro measurement. It is also possible to design a device for in vivo measurement of acoustic impedance, since the cornea could be reached directly by ultrasound without anatomic barriers. Secondly, both the anterior and posterior reflected signal of the cornea could be acquired by a highly focused transducer to calculate the impedance. Young's modulus of the anterior and posterior cornea showed a large difference in [29]. However, a smaller difference was observed in acoustic impedance in our experiment. Although cornea is composed of five layers, the innermost and outermost layers are too thin to affect ultrasound propagation, so the acoustic property of the corneal stroma is dominant. The similar anterior and posterior corneal acoustic impedance could be mutual verification to each other. Thirdly, steps should be taken to avoid or minimize the hydration of cornea during IOP elevation in vitro studies. The 20% dextran-saline solution could be used for insertion solution and immersion solution. The immersion time should be controlled to a few minutes to avoid the change of central corneal thickness.

Further studies include the following. The cornea may reach a high strain level (4%) during intraocular elevation, which could be calculated by Laplace law with the experimental parameters. The nonlinear relationship of stress and strain should be considered in the correlation between acoustic impedance and IOP. An inflation test combined with ultrasonic test should be done together, so that the elastic modulus could be calculated by shell theory model, and cornea geometry change could be recorded by laser device [14]. The strain could be calculated from ultrasonic shear wave imaging [15] and ultrasound speckle tracking [18]. The abundant data could be used to rebuild and validate the inflation model. Animal studies are expected to demonstrate more accurate correlation of corneal acoustic impedance and intraocular pressure since it is more physiologic. A low intensity level ultrasound test could be used for in vivo study to validate the possibility of intraocular pressure measurement using acoustic impedance of the cornea.

In summary, a strong correlation between intraocular pressure and acoustic impedance of the cornea was demonstrated in the present study. This correlation provided a potentially important method to noninvasively measure the intraocular pressure in vivo. This method of intraocular pressure measurement may prove useful for corneal biomechanics research as well as glaucoma clinical studies in the future.
